# Ionic mechanisms underlying atrial electrical remodeling after a fontan-style operation in a canine model

**DOI:** 10.1007/s00380-019-01544-5

**Published:** 2020-01-07

**Authors:** Jinjin Wu, Wanping Zhou, Lanping Wu, Yijiao Qian, Yanan Lu, Fen Li

**Affiliations:** 1 Cardiovascular Department, Shanghai Children’s Medical Center, Shanghai Jiaotong University School of Medicine, 1678 Dongfang Road, Shanghai, People’s Republic of China; 2 Cardiovascular Department, Children’s Hospital of Soochow University, Suzhou, Jiangsu People’s Republic of China; 3 Pediatric Cardiovascular Department, Xinhua Hospital Affiliated To Shanghai Jiao Tong University School of Medicine, Shanghai, People’s Republic of China

**Keywords:** Atrial tachycardia, Fontan operation, Atrial remodeling, Ionic remodeling

## Abstract

Atrial arrhythmia is an important cause of late death in patients after the Fontan-Style operation. However, the detailed electrophysiological characteristics of the post-Fontan atrium and its underlying mechanisms are largely unknown. In this study, we investigated electrophysiological characteristics and the ionic remodeling in the right atrium (RA) of a canine model after the Fontan operation. We performed the operation of RA to pulmonary artery connection to mimic the Fontan operation. We undertook hemodynamic measurements, cardiac electrophysiological studies, and ion current measurements. The expression of ionic channels was analyzed by PCR and western-blotting. Our Fontan model induced RA hypertension, enlarged the size of RA, and increased atrial fibrosis, representing the classic characteristic of Fontan patients. In the Fontan group, the atrial effective refractory period and the active potential duration were reduced, and the atrial tachycardia has been more often to be induced. The electrical conduction mapping showed that the Fontan group reduced the conduction velocity. The Fontan operation significantly down-regulated the expression of *KCND*3/Kv4.3, *CACNA1C*/Cav1.2 and *SCN5A*, but up-regulated the expression of *KCNJ2*/Kir2.1. Correspondingly, The Fontan operation reduced transient-outward (*I*_to_) and L-type Ca2 (*I*_Ca,L_) and *I*_Na_ currents, while increasing the inward-rectifier current (*I*_K1_). Thus, the net shortening of the action potential in the post-Fontan atrium is associated with the altered expression of ionic channels which disturbed the balance between inward and outward currents. Taken together, the Fontan operation induces the ionic remodeling, and thus altered electrophysiological characteristics of the right atrium, improving our understanding on the pathophysiology of atrial arrhythmias in Fontan patients.

## Introduction

Fontan operations or modifications are still widely used to treat patients with a tricuspid atresia (TA), a double outlet right ventricle (DORV), a double inlet left ventricle (DILV) or a single ventricle (SV) [1, 2]. The classical Fontan operation procedure is to separate systemic and pulmonary blood flow by directing a systemic venous return through the Fontan connection to pulmonary arteries and lungs without a ventricular contribution. With the development of advanced surgical techniques over the past 30 years, a growing number of these special patients have reached their adolescence and even their adulthood. Among those, atrial tachycardia (AT) is the most common early and late complication after surgical procedures [3, 4]. However, successful anti-arrhythmia drug treatments and catheter ablations are hard to be achieved. Although the reentrant activation is still considered as the main mechanism of AT after the Fontan operation [3, 4], the detailed electrophysiological characteristics of the right atrium and the exact molecular mechanism of atrial arrhythmia after the Fontan operation remain largely unknown.

Cellular electrophysiological studies have shown extensive changes in ion-channels functions leading to disturbed outward and inward currents, including a decreased transient outward K^+^ current (*I*_to_), an increased inward rectifier K^+^ current (*I*_K1_), and a decreased L-type voltage-gated Ca^2+^ current (*I*_Ca,L_), in atria from AT patients [5]. The ionic remodeling in atria disrupted the balance between inward and outward currents and changed the action potential duration and the effective refractory period, which contributed to the pathophysiology of AT [6]. Considering that the Fontan operation leads to the giant right atrium and a pressure overload of RA, we proposed that the right atrium after the Fontan operation not only caused an anatomic remodeling but also possibly induced an electrical remodeling in ion-channel functions.

In this study, we established a canine model by creating an atriopulmonary anastomosis (APA) to mimic the Fontan operation. We reported that our Fontan canine model significantly induced RA hypertension, dilation and fibrosis, which well match hemodynamic and anatomic characters of Fontan patients. Our further electrophysiological and ion channel studies revealed that the Fontan operation induced the complex ionic remodeling and changed electrophysiological characteristics of the right atrium, showing molecular mechanisms of an atrial arrhythmia after the Fontan operation.

## Materials and methods

### Animal preparation

**Fig. 1:**
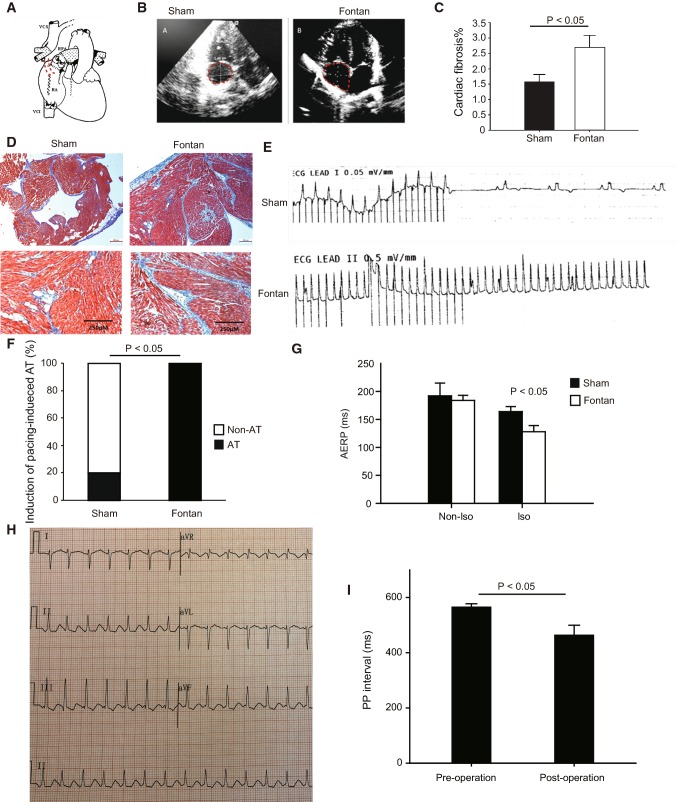
The establishment of a canine model to mimic the Fontan operation. **a** A schematic diagram of a canine model with a Fontan-style operation. The dashed line indicates the right atrium-pulmonary artery (RA-PA) connection. **b** Representative images of echocardiography of dog hearts in the sham group and the Fontan group. The dashed circle outlines the right atrium (RA). Notably, the size of RA in the Fontan group was larger than the sham group. **c**–**d** Representative images of Masson’s Trichrome staining of RA in the sham group and the Fontan group (**d**) with quantification (**c**). **e** Representative images of ECG recording in the sham group and the Fontan group. **f** The percentage of the successful induction of AT in the sham group and the Fontan group. **g** The atrial effective refractory period (AERP) recorded without and with isoprenaline in the sham group and the Fontan group. Iso, isoprenaline. **h** The limb leads of surface ECG recordings (recording speed = 25 mm/s) of a patient of pulmonary atresia and ventricular septal defect after the Fontan-style operation. It shows the sustaining atrial flutter with the atrial rate of 350 bpm. **i** P–P intervals of patients before and after the Fontan-style operation. Data are mean ± S.D. Scale bar, 250 μm

The canines, with the age around 1 year and the bodyweight 10–18.3 kg used in this study. The Fontan group, including 22 canines, received the right atrium-pulmonary artery (RA-PA) connection using a Goretex-Tunnel as well as a pulmonary valve cerclage. The sham group, including five canines, undertook the same procedures except for the RA-PA connection as the Fontan group. All canines had general anesthesia using an intravenous virbac (2–3 mg/kg, Zoletil; Schering-Plough AB, Stockholm, Sweden) and xylazine (0.1–0.2 mg/kg, Rompun; Bayer, Leverkusen, Germany) treatment as a premedication and underwent intubation with ventilator support. During the entire procedure, the limb-lead electrocardiography (ECG) and the invasive inferior arterial blood pressure (ABP) were monitored. An intravenous propofol (Recofol; Bayer Schering, Turku, Finland) treatment was used for deep general anesthesia to ensure no active motion. A systemic heparinization was administered continuously (1 mg/kg). Without a previous Glenn-procedure, a direct valveless APA was performed. An auto-pericardium graft or a 10 mm Gore-tex graft (W.L. Gore & Associates, Inc. Flagstaff, Arizona, USA.) was placed with a 6–0 continuous silk suture between the right atrial appendage and the main pulmonary artery (Fig. 1a). We were trying to block the proximal pulmonary artery as far as possible till the systolic ABP could not stay over 60 mmHg. A normal limb-ECG was performed daily and 24-h ECG recordings were made on the first postoperative day and then a week after the operation. After one week, when a second chest open was performed for electrophysiological (EP) examinations, the size of the right atrium, the tricuspid velocity, and the regurgitation as well as the pulmonary pressure were recorded as post-operative data. All animal procedures were performed in accordance with the Institutional Animal Care and Use of Laboratory Animals approved by Shanghai Jiaotong University Institutional Animal Care and Use Committee with license number SCXK-2018-0027.

### Electrophysiological protocols

The EP study for the postoperative canine was also under a continuous general anesthesia and an intubation with a ventilator support. A continuous limb-ECG was monitored. A pair of self-made platinum electrodes were put in the right atrium for stimulating RA and another electrode was fixed to the right atrial free wall for recording signals. A pacing voltage was set as the doubled pacing threshold. To induce the atrial tachycardia (AT), an atrial burst pacing at cycle lengths starting from 400 ms with a gradual decrease at a 20-ms step till an atrial ERP. When an atrial tachycardia was not inducible, a 0.3 μg/kg bolus of isoproterenol was used and then the stimulating protocol was repeated. A train of ten times 10 s-atrial bursting pacing was performed in the Fontan group and the sham group. The successful induction was considered as AT longer than 2 s. A sustained AT was defined as the AT sustaining longer than 30 s.

### Optical mapping recording

The heart was excised, and then the coronary artery was perfused ex-vivo with a Krebs solution bubbled with 95% O_2_, 5% CO_2_ at 10 mL/min flow rates and 37 °C on a Langendorff apparatus. After a 30-min stabilization and electrical–mechanical decoupling by perfusion of a Blebbistatin (Toronto Research Chemicals Inc., Ontario, Canada) solution of concentration 15 µM, the heart was loaded with a voltage-sensitive dye di-4-ANEPPS (Biotium, CA, USA, 100–150 µL, concentration 2 mg/mL of DMSO). Optical recordings were made in the right atrial region. A CCD (charge-coupled device) camera system (CardioCCD, Redshirt Imaging) recorded the potential-dependent fluorescence image at 2 k Hz [7]. A pair of bipolar electrodes was used for pacing in the right atrium close to the superior vena cava, and another pair of electrodes was used for a ventricular electrical function monitoring on the ventricles. To estimate conduction properties of the atrial preparation, optical maps were recorded during a 1.5 × threshold current of an atrial stimulation for 2 ms, at basic cycle lengths (BCLs) of 250, 200, 150, 130, 120, 110, 100, 90, 80, 70, 60, and 50 ms.

### Measurements of ion currents

The heart was initially perfused for 10 min with a Tyrode’s solution gassed with 100% O_2_; this was followed by a perfusion with a calcium-free Tyrode’s solution for 10 min. For an enzymatic digestion, the heart was perfused with a calcium-free Tyrode’s solution containing 0.475 mg/mL of collagenase (Type II, Worthington) and 1 mg/mL BSA and 0.5 mg/mL protease (Sigma, #P5147) for 60 min. Then, following an enzymatic digestion, the heart was perfused with a normal Tyrode’s solution for 5 min to remove residual enzymes. After the digestion, cells were re-suspended in a Krebs buffer. This isolation procedure typically yielded 70–90% and 40–60% rod-shaped ventricular and atrial myocytes, respectively. All myocyte electrophysiology experiments were conducted within 10 h. To determine the effective refractory period of RA (ERP), a single premature stimuli followed an 8-beat basic drive train with a pacing cycle length of 300 ms was performed in both the Fontan group and the sham group till the atrium was refractory. To measure the action potential (AP), myocytes were suspended in a small recording chamber with an external saline solution, a calcium-containing Tyrode’s solution at a bath at the temperature of 36 ± 0.5 °C. Patch pipettes were pulled from borosilicate glass (Sutter Instrument CA, USA) using a P-97 micropipette puller (Sutter Instrument CA, USA). Borosilicate glass micropipettes with a tip resistance of 1.5–3 MV, were filled with a pipette solution containing the following (in mM): 100 K-aspartate, 40 KCl, 5 MgCl_2_, 5 EGTA, 5 HEPES, pH adjusted to 7.4 with KOH. APs of cardiomyocytes were recorded in a whole-cell patch-clamp configuration using an Axopatch 200B amplifier (Axon Instruments). K^+^ currents recorded from atrial myocytes were elicited by a 100 ms pulse to + 70 mV from a holding potential of − 50 mV for *I*_to_. *I*_to_ currents were measured as the peak current amplitude. The peak *I*_K1_ current was generated by delivering 500 ms pulses to − 120 mV from a holding potential of 10 mV. *I*_Ca,L_ currents recorded from atrial myocytes were elicited by 200 ms pulses to 0 mV from a holding potential of − 70 mV delivered at 0.1 Hz. Glass pipettes were pulled from borosilicate glass (Sutter Instrument CA, USA) using a P-97 micropipette puller (Sutter Instrument CA, USA). The tip resistance of these pipettes filled with internal solutions amounted to approximately 1–2 MΩ. The pipette capacitance, the whole-cell capacitance, and the access resistance were routinely compensated. An Axopatch 200B amplifier (Axon Instruments, Foster City, CA) was used for the whole-cell voltage clamping. Voltage clamp pulse delivery and data acquisition were controlled by an IBM PC running pClamp software (Axon Instruments). After rupture of the cell membrane to enter the whole-cell mode, the current amplitude and kinetics were allowed to stabilize for 3–7 min before initiating the experimental procedure. *I*_Na_ currents were recorded in a solution containing MgCl_2_ 1.5 mM, TEA 80 mM, CaCl_2_ 1.8 mM, CsCl 5 mM, HEPES 20 mM, Glucose 11 mM, 4-aminopyridine 3.0 mM and MnCl_2_ 2.0 mM, adjusted to a pH of 7.4. For K currents, the ionic composition of the water solution used to atrial myocytes (external solution) for recording I_to_: a calcium-free Tyrode’s buffer containing CaCl_2_ 1 mM, CdCl_2_ 200 nM, Atropine 200 nM, Tetraethylammonium 10 mM, adjusted to a pH of 7.4 with NaOH. The ionic composition of the water solution used to atrial myocytes (an external solution) for recording *I*_K1_: a calcium-free Tyrode’s buffer containing CaCl_2_ 1 mM, CdCl_2_ 200 μM, Atropine 200 nM, 4-aminopyridine 2 mM, BaCl_2_ 1 mM. The ionic composition of the internal solution of the patching pipette for both *I*_to_ and *I*_K1_ was (in mM): K-aspartate 110 mM, KCl 20 mM, MgCl_2_ 1 mM, ATP 5 mM, Li-GTP 0.1 mM, HEPES 10 mM, Na-phosphocreatine 5 mM, EGTA 5 mM, pH = 7.3 with KOH. For calcium currents, Ca currents (*I*_Ca,L_) were measured in atrial myocytes using an external solution of the following composition (in mM): TEA 136 mM, CsCl 5.4 mM, CaCl_2_ 2 mM, MgCl_2_ 0.8 mM, HEPES 10 mM, Dextrose 10 mM, Niflumic Acid 50 μM, pH = 7.3 with CsOH, whereas the solution used to fill the pipette had the following ionic solution (in mM): CsCl 120 mM, TEA 20 mM, MgCl_2_ 1 mM, ATP 5 mM, LiGTP 0.1 mM, EGTA 10 mM, HEPES 10 mM, adjusted to pH 7.4 with CsOH.

### Quantitative RT-PCR

Quantitative real-time PCR was performed using a Maxima™ SYBR Green PCR Master Mix (Fermentas) and an ABI PRISM 7700 Sequence Detection System according to the manufacturer’s instructions. The datum was normalized to the mRNA level of GADPH.

### Western-blotting analyses

Total protein extracts (30 g) from the heart atrium were resolved on SDS-PAGE gels and transferred to PVDF membranes for western-blotting. Antibodies against Kv4.3(SANTA CRUZ, USA, C-17), Kir2.1 (SANTA CRUZ, C-20), Cav1.2 (SANTA CRUZ, G-14), and β- actin (Sigma, USA) were used.

### Histological analysis

The 5-μm-thick paraffin-embedded sections were stained with hematoxylin and eosin and Masson Trichrome (MT). The area of fibrosis in myocardium was calculated by imageJ.

### ECG recordings of patients who underwent the Fontan-style operation

The surface ECG recordings of 16 patients (single ventricle *n* = 7; pulmonary atresia and ventricular septal defect *n* = 6; double outlet right ventricle *n* = 3) who underwent Fontan-style operations were documented and analyzed. The cardiac rhythm and the P–P interval (P-peak to P-peak) were measured in the ECG (recording speed of 25 mm/s). If the patient had an atrial flutter, the F–F interval (F-peak to F-peak) was measured instead of the P–P interval, which means the cycle length of the atrial activation. Then, the cardiac rhythm and the P–P interval were compared before and 7 days after the Fontan operation.

### Statistical analysis

Continuous data are expressed as mean ± standard deviation (mean ± SD). Two independent groups were compared using the two-tailed Student’s *t* test and the Fisher’s exact test for categorical data. A one-way ANOVA analysis was used for multiple comparisons. All data were tested for normality and equal variance before using parametric tests. All analyses were performed with SPSS 11.0 (SPSS. Inc) for Windows. *P* < 0.05 was considered statistically significant.

## Results

### Hemodynamic indices of animals after the successful Fontan-style operation

We performed the right atrium-pulmonary artery (RA-PA) connection procedure in all 22 canines (Fig. 1a). In the Fontan group, five dogs survived till 7 days after the Fontan-style operation, and others survived shorter than one week because of an acute heart failure or a severe ventricular arrhythmia. In the sham group, all five dogs survived until their scarification. We performed the hemodynamic measurement in the Fontan group (*n* = 5) and the sham group (*n* = 5), as shown in Table 1. Compared with the sham group (121.2 ± 8.78 bpm), heart rates (HRs) were significantly increased in the Fontan group (141.8 ± 10.11 bpm, *P* < 0.05). The catheter examination showed that the right atrium pressure in the Fontan group (18.4 ± 2.51 mmHg) was significantly higher than the sham group (10.6 ± 0.89 mmHg, *P* < 0.05). The pulmonary artery pressure was similar between the sham group and the Fontan group (12.6 ± 2.41 mmHg vs. 13.0 ± 2.74 mmHg, respectively) (Table 1). All the above hemodynamic changes in the Fontan canine group matched hemodynamic characteristics of Fontan patients [8], suggesting that this canine model could mimic the clinical Fontan operation.

**Table 1:** Hemodynamic characteristics

No	Sham group					Fontan operation				
	HR (bpm)	RA (mm)	RAP (mmHg)	PAV (m/s)	PAP (mmHg)	HR (bpm)	RA (mm)	RAP (mmHg)	PAV (m/s)	PAP (mmHg)
1	124	14	12	1.0	11	145	18	18	0.6	16
2	134	14	10	0.8	12	156	18	15	0.8	15
3	110	15	10	1.0	16	140	16	18	0.7	12
4	118	12	11	0.8	14	128	16	19	0.8	9
5	120	15	10	0.8	10	140	20	22	0.6	13
mean	121.2	14	10.6	0.88	12.6	141.8*	17.6*	18.4*	0.7*	13
SD	8.78	1.22	0.89	0.11	2.41	10.11	1.67	2.51	0.10	2.74

*HR*  heart rate, *PA* pulmonary artery, *PAV*  pulmonary artery velocity, *RA*  right atrium, *RAP*  right atrium pressure

*P < 0.05

The 24-h ECG recording showed that non-sustaining atrial tachycardia (NSAT, AT sustaining shorter than 30 s) was observed in 3 of 5 canines in the Fontan group, while no observation in the sham group. We also collected clinical data of 16 patients who underwent Fontan operations and compared the cardiac rhythm and the P–P interval of these patients before and 7 days after Fontan operations. We observed that P–P intervals of patients after Fontan operation was shorter than those before the operation (465.63 ± 139.98 ms vs. 567.19 ± 42.18 ms, p = 0.016) (Fig. 1i). Sustaining atrial tachycardia was noted in two patients in the first week after the operation (Fig. 1h). One had atrial tachycardia and the other had atrial flutter (Fig. 1h). Our results supported that this canine model after the Fontan-style operation represents proarrhythmic propensity in Fontan patients.

### The Fontan operation induced anatomical remodeling and cardiac fibrosis

Echocardiography showed that the right atrium was larger in the Fontan group (17.6 ± 1.67 mm) than that in the sham group (14.0 ± 1.22 mm, *P* < 0.05) (Fig. 1b; Table 1) observed in Fontan patients [8]. The mean wall thickness of the right atrium in Fontan hearts was 2.4 ± 0.5 mm, thicker than the sham group, although not significantly (1.8 ± 0.3 mm). Our further histological analysis detected more severe atrial fibrosis in the Fontan RA in comparison with the sham group (Fig. 1c–d), as shown by the Masson’s Trichrome staining. This well matched atrial fibrosis in Fontan patients [8].

### In vivo electrophysiology of the right atrium after the Fontan operation

**Fig. 2:**
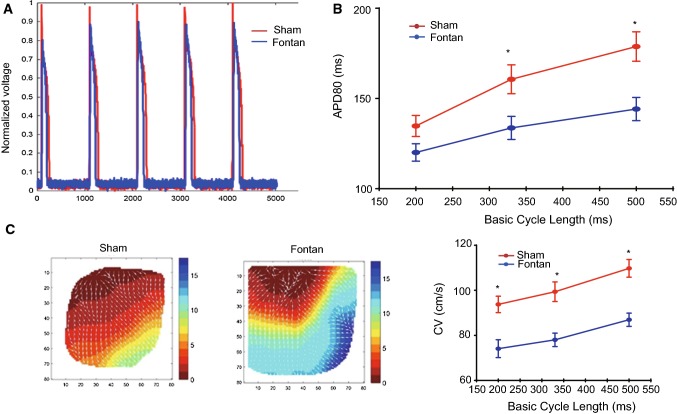
In vivo electrophysiology of the right atrium after the Fontan operation. **a**–**b** Atrial action potential measurements and the quantification of APD80 in the indicated RA. **c** The electrical conduction mapping and the quantification of conduct velocity (CV) in RA of the sham group or the Fontan group. Data are mean ± S.D. *P < 0.05

Under the atrial burst stimulation with the cycle length of 200 ms, the induction of sustaining atrial tachycardia was noted in all five Fontan canines but only in one control canine (Fig. 1e–f). Although atrial effective refractory period (AERP) in the Fontan group and in the sham group was comparable, AERP in the Fontan group was significantly shorter than the control group upon an isoprenaline stimulation (Fig. 1g). The active potential duration (APD) in the Fontan group was significantly shorter than that in the sham group (Fig. 2a–b; Table 2). Our data showed that the Fontan operation resulted in a significant reduction in the upstroke velocity (dV/dt) by 9.7 ± 3.6% (mean ± SD) leading to a decrease in phase 0 amplitude and in APD80. The electrical conduction mapping showed that the conduction velocity (CV) significantly decreased in the Fontan group at all basic cycle lengths (BCLs) (Fig. 2c; Table 2).

**Table2:** RA electrical remodeling after Fontan operation

	Sham group			Fontan group		
BCL	200 ms	330 ms	500 ms	200 ms	330 ms	500 ms
APD80 (ms)	134.77 ± 21.04	160.62 ± 28.79	178.77 ± 29.43	120.08 ± 17.40*	133.69 ± 23.08*	144.15 ± 23.15*
CV (cm/s)	93.74 ± 13.17	99.35 ± 15.69	109.71 ± 14.19	74.08 ± 14.29*	78.00 ± 10.17*	86.92 ± 10.59*
CPHI	2.54 ± 0.67	2.54 ± 0.67	2.49 ± 0.75	3.16 ± 0.64*	3.16 ± 0.64*	3.02 ± 0.22*

APD action potential period, BCL basic cycle length, CPHI conduction phase heterogeneity index, CV conduction velocity, RA right atrium

*P < 0.05

### The Fontan operation reduced *I*_to_ currents in atrial cardiomyocytes

**Fig. 3:**
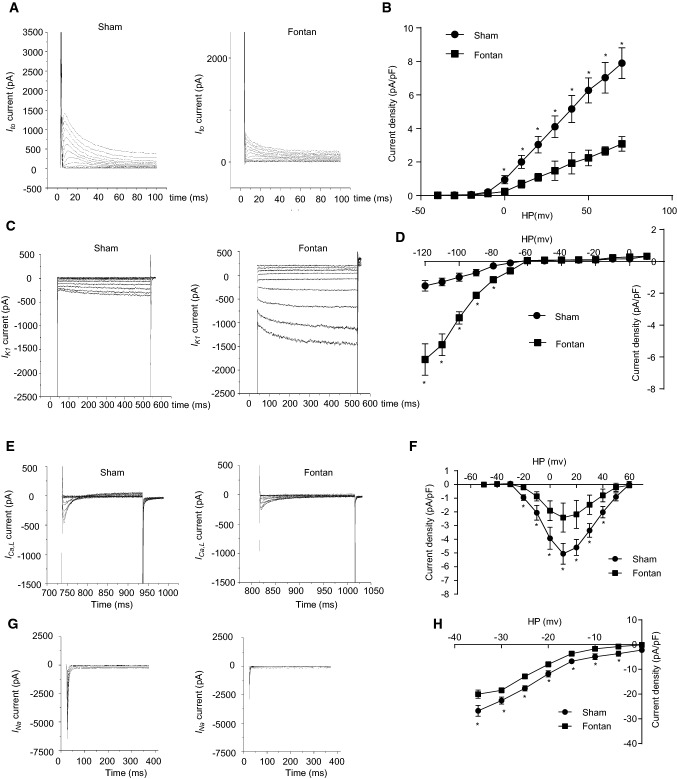
Cellular electrophysiological study of ion currents in RA after the Fontan operation. **a***I*_to_ current measurements in atrial cardiomyocytes in the indicated group. **b***I*_to_ density measurements in atrial cardiomyocytes of the indicated group. **c***I*_K1_ current measurements in atrial cardiomyocytes in the indicated group. **d***I*_K1_ density measurements in atrial cardiomyocytes of the indicated group. **e***I*_Ca,L_ current measurements in atrial cardiomyocytes in the indicated group. **f***I*_Ca,L_ density measurements in atrial cardiomyocytes of the indicated group. **g***I*_Na_ current measurements in atrial cardiomyocytes in the indicated group. **h** *I*_Na_ density measurements in atrial cardiomyocytes of the indicated group. Data are mean ± S.D. *P < 0.05

**Fig. 4:**
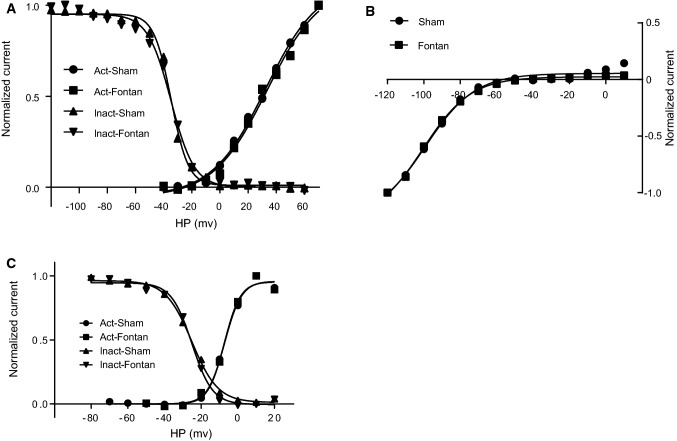
Cellular electrophysiological study of ion currents in RA after the Fontan operation. **a** The voltage dependence of *I*_to_ inactivation and activation in atrial cardiomyocytes of the indicated group. **b** The voltage dependence of *I*_K1_ activation in atrial cardiomyocytes of the indicated group. **c** The voltage dependence of *I*_Ca,L_ inactivation and activation in atrial cardiomyocytes of the indicated group

Previous investigations showed that atrial fibrillation induced a reduction of the transient outward current (*I*_to_) contributing to the pathogenesis of atrial tachycardia [9]. We next recorded *I*_to_ currents using the whole-cell patch-clamp to examine whether the Fontan operation disturbed *I*_to_ currents. RA cardiomyocytes from the Fontan group displayed lower *I*_to_ currents than the sham group (Fig. 3a). The *I*_to_ density was significantly reduced in the Fontan group compared with the sham group (Fig. 3b). The *I*_to_ activation and inactivation were not affected by the Fontan operation (Fig. 4a).

### The Fontan operation increased atrial *I*_K1_ currents

It has been previously reported that the increased inward rectifier potassium current, *I*_K1_*,* contributed to AT-induced electrical remodeling and the self-perpetuating nature of AF [5, 10]. We next investigated the change of *I*_K1_ in the Fontan canine model. In contrast to *I*_to_ currents, *I*_K1_ currents were significantly increased in the Fontan group compared with the sham group (Fig. 3c). The *I*_K1_ density was higher in the Fontan group than the sham group (Fig. 3d). The activation curve of *I*_K1_ showed no differences between the sham group and the Fontan group (Fig. 4b).

### Reduced *I*_Ca,L_ currents in RA after the Fontan operation

Atrial L-type Ca currents (*I*_Ca,L_) have been shown to decline in heart diseases, contributing to the pathogenesis of AT [5, 10]. We determined whether *I*_Ca,L_ was affected in post-Fontan atrial cardiomyocytes. Our cellular electrophysiological studies showed that *I*_Ca,L_ currents were significantly reduced in the Fontan group versus the sham group (Fig. 3e). The Fontan group displayed significantly lower *I*_Ca,L_ density than the sham group (Fig. 3f). The *I*_Ca,L_ activation and inactivation were not affected by the Fontan operation (Fig. 4c).

### Reduced *I*_Na_ currents in RA after the Fontan operation

Atrial sodium currents (*I*_Na_) have been shown to affect the RA conduction and contribute to the pathogenesis of AT [11, 12]. We examined whether *I*_Na_ currents were affected in post-Fontan atrial cardiomyocytes. Our cellular electrophysiological studies showed that *I*_Na_ currents were significantly reduced in the Fontan group versus the sham group (Fig. 3g). The Fontan group displayed significantly lower *I*_Na_ density than the sham group (Fig. 3h). These results showed that Fontan-induced a reduction in *I*_Na_ currents might contribute to the slower upstroke velocity of AP and affect the RA conduction velocity, promoting arrhythmia after the Fontan operation.

### Down-regulation of Kv4.3/*KCND3*, Cav1.2/*CACNA1C* and* SCN5A* expression and up-regulation of Kir2.1/*KCNJ2* expression in the right atrium of the Fontan model

**Fig. 5:**
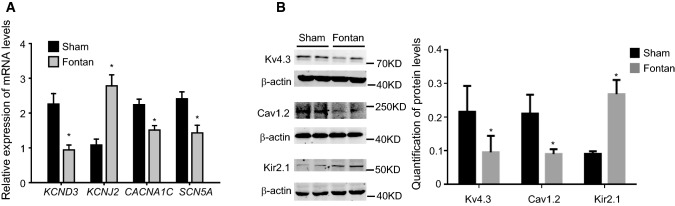
Down-regulation of Kv4.3/*KCND3* and Cav1.2/*CACNA1C* expression and up-regulation of Kir2.1/*KCNJ2* expression in the right atrium of the Fontan model. **a** Relative expression levels of *KCND3*, *CACNA1C*, *KCNJ2* and *SCN5A* mRNA in the indicated RA quantified by quantitative real-time PCR. **b** Western-blotting analysis of Kv4.3, Kir2.1 and Cav1.2 protein levels with their quantification. Data are mean ± S.D. *P < 0.05

It has been shown that ion channel Kv4.3 (encoded by *KCND3*), Kir2.1 (encoded by *KCNJ2*), and Cav1.2 (encoded by *CACNA1C*), are the primary determinants of *I*_to_, *I*_K1_ and *I*_ca,L_ currents. We hypothesized that the Fontan operation might alter the expression of Kv4.3/*KCND3*, Kir2.1/*KCNJ2*, Cav1.2/*CACNA1C*, and *SCN5A* and thus changed *I*_to_, *I*_K1_,* I*_ca,L_ and *I*_Na_ currents, respectively. As expected, our data showed that Kv4.3/*KCND3* was down-regulated in the RA of the Fontan group (Fig. 5a–b), which might contribute to the reduced *I*_to_ current and density. The Fontan operation up-regulated the expression of Kir2.1/*KCNJ2* in RA (Fig. 5a–b), which might explain that the increased *I*_K1_ currents in Fontan hearts. The reduction of Cav1.2/*CACNA1C* in RA parallels with the reduced *I*_ca,L_ currents in atrial cardiomyocytes of the Fontan group (Fig. 5a–b), suggesting Fontan-induced down-regulation of Cav1.2/*CACNA1C* is associated with the change in *I*_ca,L_ currents. The Fontan-style operation down-regulated the expression of *SCN5A* in the right atrium, which might contribute to the reduced *I*_Na_ currents in the Fontan group. Thus, our data suggested that the Fontan operation resulted in changes in Kv4.3/*KCND3*, Kir2.1/*KCNJ2*, Cav1.2/*CACNA1C*, and *SCN5A*, which may contribute to ionic remodeling and proarrhythmia propensity after the Fontan operation.

## Discussion

The Fontan procedure can offer an effective hemodynamic palliation for most univentricular patients, but problematic atrial tachycardia is the most common severe complication. In this study, we reported a successful Fontan canine model, allowing us to examine both post-Fontan anatomical and electrical remodeling in more detail. Our results showed that the Fontan operation enlarged the size of the right atrium and increased atrial fibrosis and suggested that the anatomical remodeling could be one of mechanisms underlying post-Fontan atrial arrhythmia. The macroreentry relating to suture lines or/and other conduction obstacles was still known as the main mechanism for atrial tachycardia after Fontan procedures [3, 4, 13, 14], and focal atrial tachycardia or localized reentry tachycardia has also been reported in Fontan patients [13, 14]. However, molecular mechanisms underlying Fontan-induced atrial arrhythmia remain obscure.

It has been well known that the atrial electrophysiological remodeling was an important contributor to atrial arrhythmia. The atrial tachycardia-induced remodeling shows changes in atrial electrophysiological characteristics associated with up- or down-regulation in multiple ionic channels. Previous investigations reported that the transient outward currents (*I*_to_) decreased in the patients with atrial fibrillation and that down-regulation of *KCND3*/Kv4.3 expression contributed to the reduction of *I*_to_ currents, increasing the susceptibility for atrial tachycardia [9]. In the pathogenesis of heart failure, the ion channel remodeling of the reduced *I*_to_ currents was also observed in the right atrium [15]. In line with the observations from atrial fibrillation and heart failure, we also detected a down-regulation of *KCND3*/Kv4.3 expression and the decrease in *I*_to_ currents, which might be an important contributor to post-Fontan atrial arrhythmia.

The density of *I*_K1_ among different cardiac tissue types is highly variable, large in the ventricle and small in the atrium. As the inward rectifier potassium currents, the increased *I*_K1_ currents have been reported to be associated with the onset of AF [5]. *KCNJ2*/Kir2.1 is an important contributor to *I*_K1_ currents, and the change in *KCNJ2*/Kir2 expression influences *I*_K1_ currents [16]. In our study, we detected the up-regulation of *KCNJ2*/Kir2 expression induced by the Fontan operation, explaining the increased *I*_K1_ currents in the Fontan atrium. Kharche S et al. reported that the increased *I*_K1_ currents influenced the atrial conduction velocity and promoted the atrial susceptibility to arrhythmia [17].·In consistence with this report, our investigation revealed the increased *I*_K1_ currents in RA after the Fontan-style operation might contribute to alter the RA conduction velocity. Moreover, we detected a reduction of *SCN5A* expression and correspondingly resulted in the decreased *I*_Na_ currents in the RA after the Fontan operation, which contributed to retard the RA conduction and promoted atrial arrhythmia. Indeed, we detected more frequent atrial tachycardia induced by the atrial burst pacing, suggesting that the *I*_K1_ and *I*_Na_ remodeling might contribute to proarrhythmia propensity after the Fontan operation.

The most important ionic change is a decrease in L-type Ca^2+^ current, which reduces APD and APD adaptation to rate [18]. The reduced expression of *CACNA1C*/Cav1.2 (*I*_Ca,L_) has been shown to be associated with heart diseases, including atrial fibrillation [19–21], ventricular tachycardia [22], and dilated cardiomyopathy, contributing to the pathogenesis of arrhythmia. In this study, we provided in vivo and ex vivo evidence to show that the Fontan operation induced the down-regulation of *CACNA1C*/Cav1.2 expression and, therefore, reduced *I*_Ca,L_ currents, producing an electrophysiological substrate for the development of atrial arrhythmia.

In our study, most of 22 canines died in 3 days after the Fontan-style operation because of acute heart failure or severe ventricular arrhythmia. Only five dogs survived till 7 days after the Fontan-style operation, and others survived shorter than one week because of acute heart failure or severe ventricular arrhythmia. No canine could survive until 30 days in our investigation. Because of an extremely low survival rate of dogs after the Fontan-style operation, it was too difficult to investigate cardiac remodeling more than 1 week after the RA-PA connection in our experiments. Despite this limitation, our data have already shown significant anatomical changes and the electrical remodeling one week after the Fontan-style operation, suggesting that one week was long enough to induce the RA remodeling upon the RA-PA connection in canines. Except for *I*_K1_ and *I*_to_ currents, we did not examined other potassium currents including ligand-gated [ATP-sensitive (*I*_K, ATP_)*,* acetylcholine-activated (*I*_K, Ach_)*,* and Ca^2+^-activated (*I*_K, ATP_)] K^+^ currents and delayed rectifier K^+^ currents (*I*_Kur_, *I*_Kr_, and *I*_Ks_), which controlled the resting membrane potential, the action potential duration and the refractoriness in cardiomyocytes. This limitation in our study cannot exclude the possibility that other potassium currents might be involved in the electrical remodeling in RA after the Fontan-style operation. We will examine more detail in K^+^ currents in RA after the Fontan operation in our future research.

## Conclusions

We first reported in vivo electrophysiology of the right atrium and the detailed cellular electrophysiology of atrial cardiomyocytes after the Fontan operation. Our electrophysiological studies revealed that Fontan operation resulted in complex ionic remodeling caused by the aberrant expression of ionic channels, including Kv4.3/*KCND3* (*I*_to_), Kir2.1/*KCNJ2* (*I*_K1_), Cav1.2/*CACNA1C* (*I*_Ca,L_), and *SCN5A* (*I*_Na_), which might account for the net shortening of AERP and APD in the post-Fontan atrium as an important arrhythmogenic substrate for atrial tachycardia. Together, besides the Fontan operation-induced anatomical remodeling, the complex electrical remodeling essentially contributes to the development of atrial arrhythmia, with a potentially important implication for understanding post-Fontan arrhythmia mechanisms and improving therapy.
